# Polymeric Membranes for Oil-Water Separation: A Review

**DOI:** 10.3390/polym14050980

**Published:** 2022-02-28

**Authors:** Evgenia S. Dmitrieva, Tatyana S. Anokhina, Eduard G. Novitsky, Vladimir V. Volkov, Ilya L. Borisov, Alexey V. Volkov

**Affiliations:** Polymeric Membrane Laboratory, A.V. Topchiev Institute of Petrochemical Synthesis, Russian Academy of Sciences, Leninsky pr., 29, 119991 Moscow, Russia; dmitrievaes@ips.ac.ru (E.S.D.); ednov@ips.ac.ru (E.G.N.); vvvolkov@ips.ac.ru (V.V.V.); boril@ips.ac.ru (I.L.B.); avolkov@ips.ac.ru (A.V.V.)

**Keywords:** oil-water emulsions, commercial polymers, polymeric membranes, polyvinylidene fluoride, polyacrylonitrile, polysulfone, membrane separation, membrane modification, pollution resistance

## Abstract

This review is devoted to the application of bulk synthetic polymers such as polysulfone (PSf), polyethersulfone (PES), polyacrylonitrile (PAN), and polyvinylidene fluoride (PVDF) for the separation of oil-water emulsions. Due to the high hydrophobicity of the presented polymers and their tendency to be contaminated with water-oil emulsions, methods for the hydrophilization of membranes based on them were analyzed: the mixing of polymers, the introduction of inorganic additives, and surface modification. In addition, membranes based on natural hydrophilic materials (cellulose and its derivatives) are given as a comparison.

## 1. Introduction

**Figure 1:**
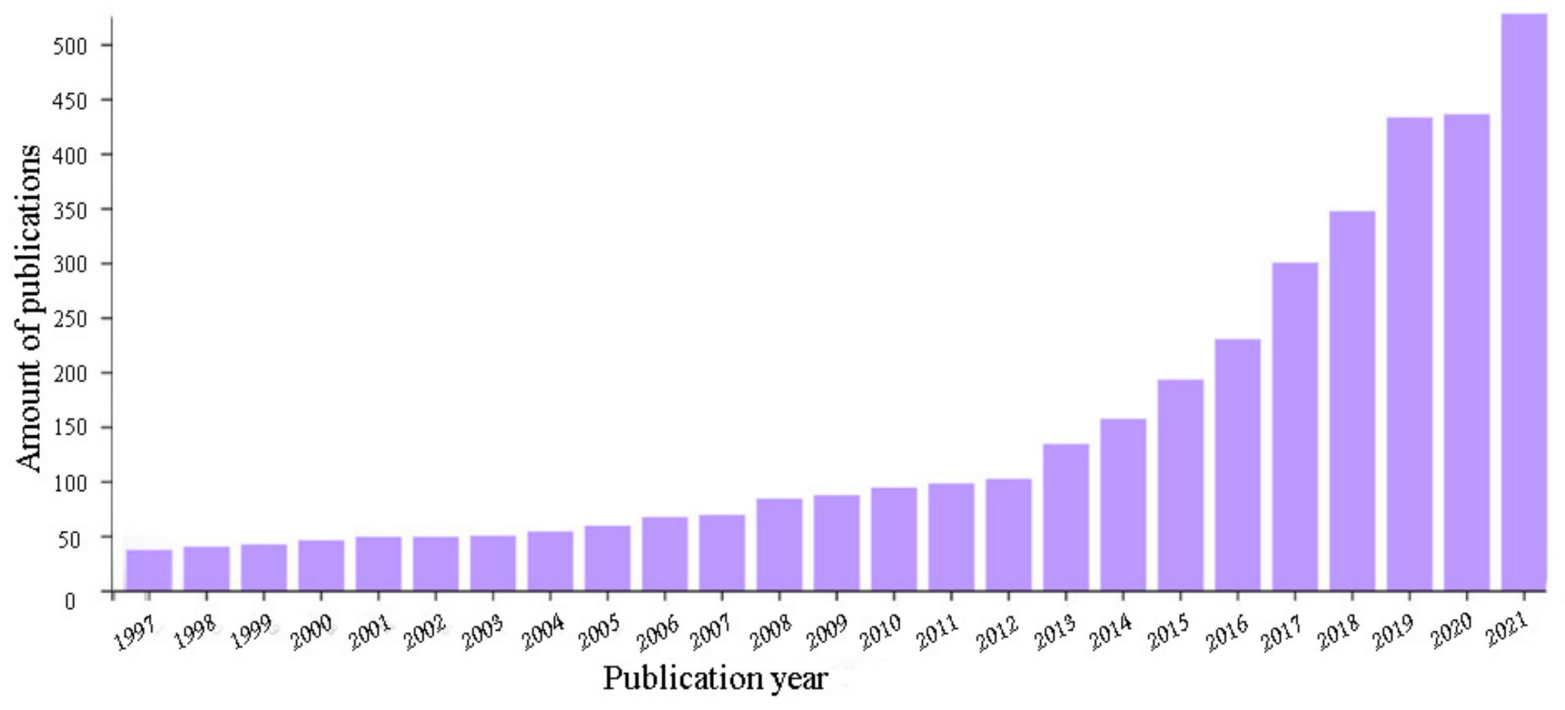
The number of publications in the field of membrane separation of oil-water emulsions in the Web of Science collection over the past 25 years (search was performed using the keywords “membrane + oil water emulsion”).

The exploration, production, transportation, and refining of crude oil are always associated with the separation of water-oil mixtures as the primary stage of oil refining or due to the accidents resulting in the oil spilling. In the case of oil-polluted surface waters, there are a big variety of adsorbents traditionally used to collect the excess of the oil, including sponges [1,2], foam materials [3], and natural adsorbents [4]. However, the complete cleaning of water from the presence of the oil is complicated by the formation of oil-water emulsions, which become more stable due to the presence of different components in the oil or to water acting as emulsifiers such as asphaltenes, resins, mineral salts, clay particles, etc. [5]. To overcome this problem, a number of methods have been developed to breakdown such stable oil-water emulsions: chemical [6,7], biological [8,9], mechanical (cyclones, separators, settling tanks, centrifuges) [10,11], thermal [12,13], microwave [14,15], electrical [16,17], ultrasonic [18,19], and membrane separation [20,21]. Considering the last one, it can be seen from Figure 1 that the number of publications on the application of membrane for the separation of the oil-water emulsion has been increased by a factor of five during the last ten years.

Based on the size of the oil droplets in water, the following classification of the oil phase can be used: free oil (>150 µm), dispersed oil (20–150 µm), and emulsified oil (<20 µm) [22]. The membrane filtration is considered a promising technology for separating oil droplets with a size smaller than ~10 µm [21]. Thus, ultrafiltration (UF) and tighter microfiltration (MF) are the more relevant choices for the separation of the oil droplet size range of 1–10 µm from the water [23] and are attracting increased attention [24,25].

The effective purification of water from finely dispersed emulsions is important due to the constant tightening of regulatory requirements for the concentration of petroleum products in discharged process waters. For instance, in the United States, the maximum daily concentration of petroleum products in waters discharged into the sea is set at 42 ppm [26], whereas The Oslo Paris Convention (OSPAR Convention) has reduced the maximum content of petroleum products in produced water discharged into the sea to 30 ppm [27]. In addition, the absence of phase changes during the filtration and the low size of the membrane units can significantly save energy and footprint, reducing the cost of purified water [28]. It has been calculated that the payback periods for oil-water emulsion treatment plants by evaporation, electrocoagulation, and ultrafiltration methods were 1.14, 0.89, and 0.22 years, respectively [24].

**Scheme 1:**
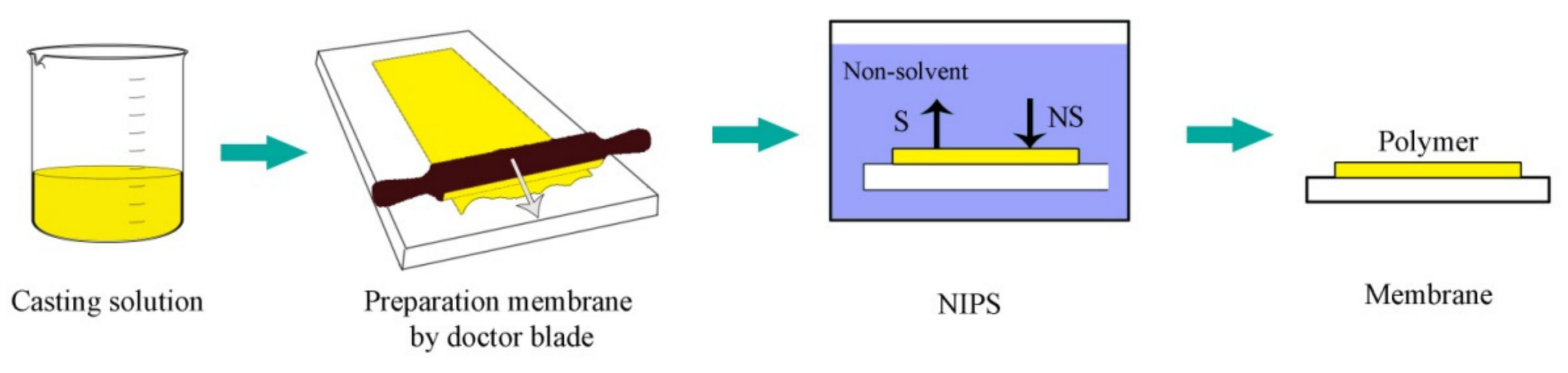
Scheme of membrane formation by the NIPS method: P—polymer, S—solvent, NS—non-solvent.

The membranes show a number of advantages over other methods such as the high efficiency of separation of fine water-oil emulsions, the absence of additional chemicals, high separation capacity, energy efficiency, compactness, and modularity of equipment. However, its further application is partially hindered by the high capital costs required for large volumes of effluent and the fouling/degradation of the polymeric membranes during long-term operation [29,30]. The commercial UF and MF membranes are typically fabricated from a number of bulk polymers [31,32,33], particularly polysulfone (PSf), polyethersulfone (PES), polyvinylidene fluoride (PVDF), and polyacrilonitrile (PAN) [34,35]. The porous structure of MF/UF membranes is formed as a result of phase inversion of polymeric solution [36]: thermally induced phase separation (TIPS) [37,38], evaporation induced phase separation (EIPS), vapor induced phase separation (VIPS) [39,40], non-solvent induced phase separation (NIPS) [36], or a combination of these methods. The most common is the NIPS method, when the polymeric solution is separated into two phases, polymer-rich and solvent-rich ones, due to the interaction with non-solvent (typically, water) once the formed polymeric film is in the precipitation bath [41]. The schematic illustration of the formation of the porous membrane by the NIPS method is given in Scheme 1.

**Figure 2:**
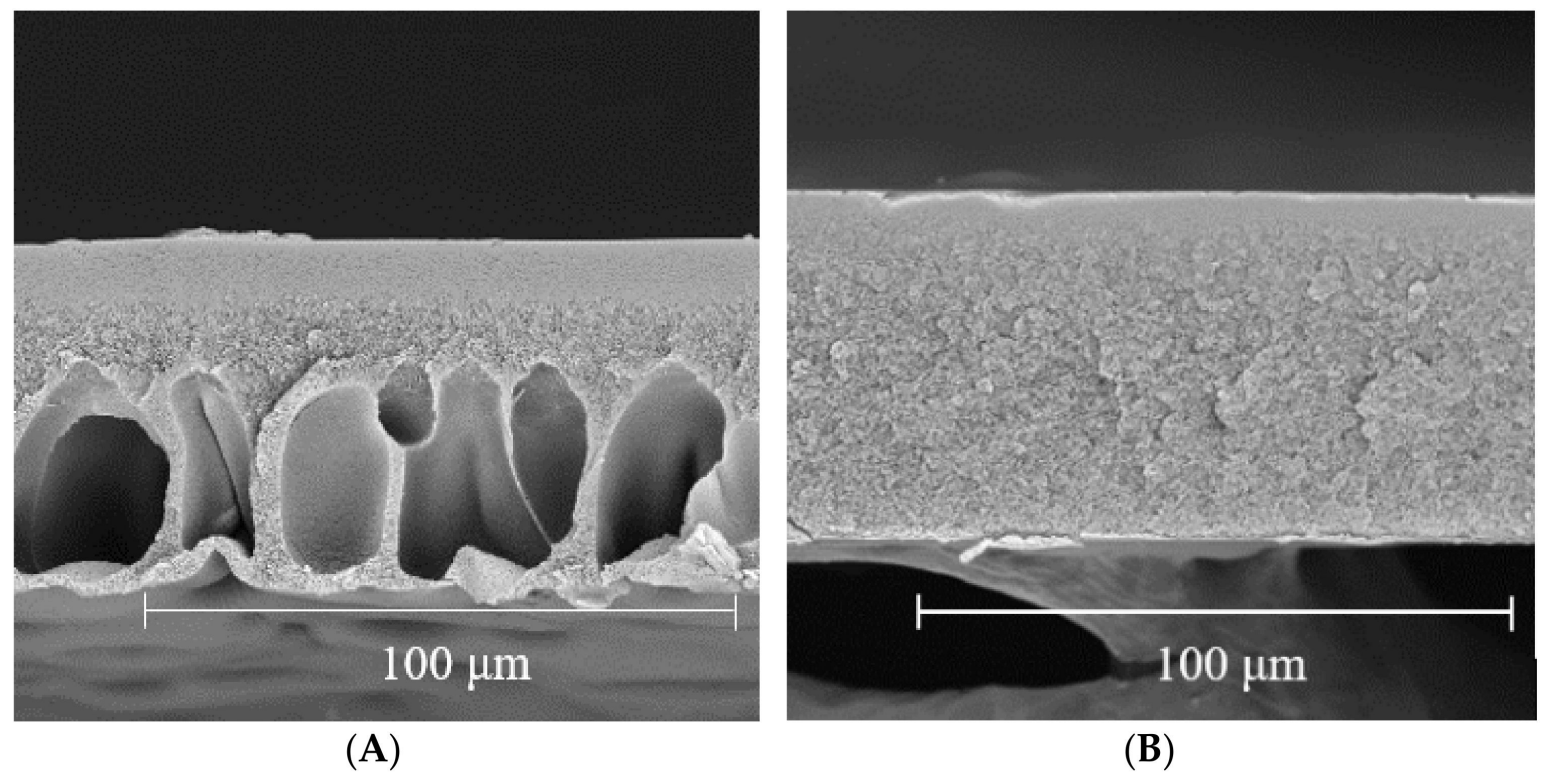
SEM micrographs of the cleavage of flat membranes obtained from a solution of PAN in DMSO: (**A**) 13 wt.%, (**B**) 15 wt.% [42].

The NIPS method allows for the processing of the majority of bulk polymers into the filtration membranes. By adjusting the parameters of membrane formation such as polymer concentration, solvent and non-solvent nature, and the presence of various additives, it is possible to control the porous structure of the filtration membranes. As can be seen from Figure 2, it was possible to change the porous structure of PAN membranes from finger-like to sponge-like by increasing polymer concentration in the casting solution from 13 up to 15 wt.%.

However, the majority of synthetic polymers considered above are semi-hydrophobic materials; for instance, the water contact angles for PSf, PES, PVDF, and PAN are 84 [43], 79 [43], 120 [44], and 100°, respectively. Because of their hydrophobicity, the traditional polymeric membranes suffer from oil fouling, which includes blocking the membrane pores, reducing the water flux, decreasing the membrane lifespan, and deteriorating product water quality with increasing energy consumption [30].

Numerous studies have shown that membranes with superhydrophobic and superoleophobic surfaces demonstrate the best resistance towards oil pollution [30]. Such phenomena can be explained by the formation of a dense hydrated layer on the membrane surface, which reduces the direct contact and, hence, the adhesion of pollutants to the polymer [45,46]. Thus, the hydrophilization of the surface and/or bulk of the membrane can be considered as a perspective approach to reduce the contamination of commercial membranes made of bulk polymers. Bearing this in mind, this review is devoted to the production of membranes for the separation of oil-water emulsions based on commercial synthetic polymers (PSf, PES, PVDF, PAN) by the phase-inversion method. Various approaches to the hydrophilization of filtration membranes made of PSf, PES, PVDF, and PAN are discussed in order to increase their separating ability and pollution resistance. In addition, some focus on the application of natural polymers is also given due to current trends in “green” chemistry and sustainable developments.

## 2. Commercial Polymer Membranes

**Figure 3:**
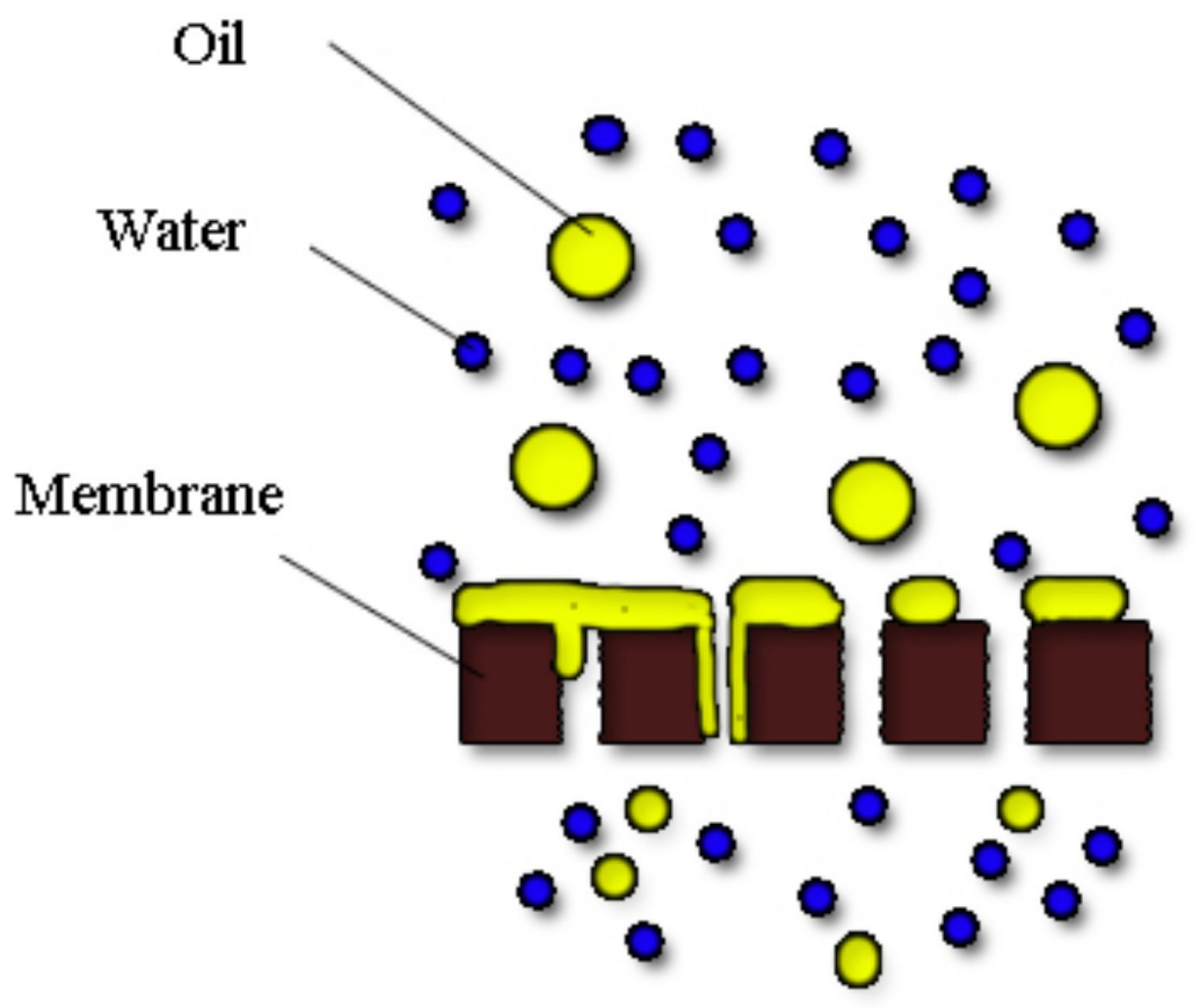
Simplified depiction of oil-water emulsion separation and membrane fouling.

Most commercial polymers are hydrophobic, which causes them to be prone to contamination when separating oil-water emulsions (Figure 3). To eliminate this disadvantage, various methods of modification can be used including physical (which does not cause the formation of new substances and chemical bonds) [47] or chemical, in which new chemical bonds appear on the surface and/or in the volume of membranes [48,49,50,51].

**Figure 4:**
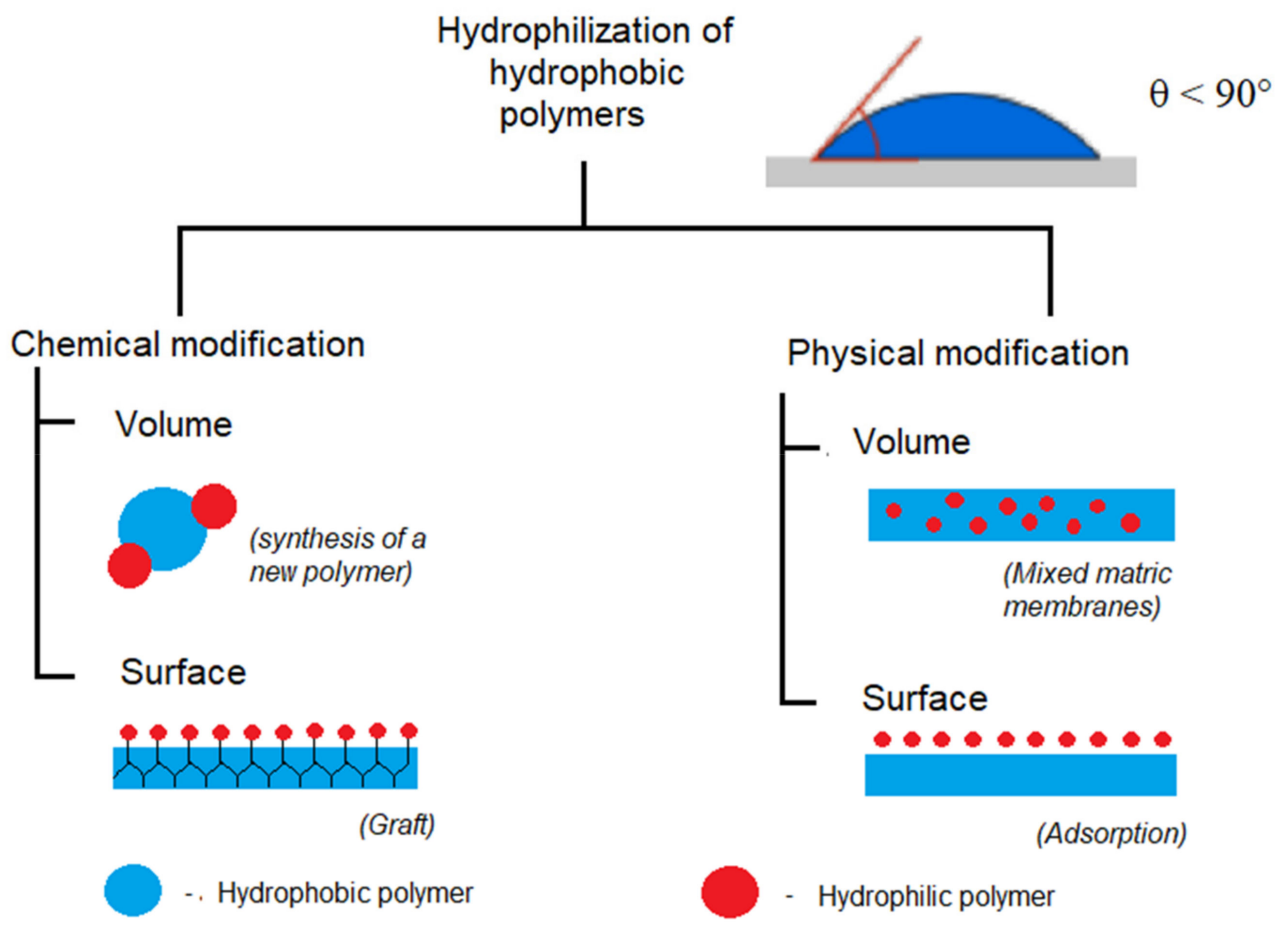
General approaches to the hydrophilization of hydrophobic polymers.

Figure 4 represents the schematic overview of hydrophilization methods to reduce the water contact angle [45,46].

Chemical bulk modification can be viewed as the creation of a new polymer, which is usually achieved by a copolymerization reaction. This method makes it possible to achieve a sharp change in membrane properties but is rather complicated.

Chemical surface modification-grafting of hydrophilic functional groups has several advantages over other modification methods [52]:

- Ability to control the process and change the desired grafting density by changing the concentrations and time;
- Precise localization of functional groups on the surface;
- Long-term chemical stability, which is provided by the covalent attachment of functional groups.

The physical bulk modification involves the formation of membranes for the separation of water-oil emulsions using mixtures of synthetic hydrophobic polymers (for example, polysulfone (PSf)) and hydrophilizing additives (for example, acetates [53], polyethylene glycol [54], polyvinylpyrrolidone [55] or particles of inorganic substances [56,57]). It should be noted that these techniques equally apply to both flat and hollow fiber membranes. It is believed that this modification method is one of the most effective due to its simple implementation and the good reproducibility of the results [58].

The physical surface modification consists in applying thin selective layers of hydrophilic polymers and/or inorganic particles. The methodology involves the deposition of a hydrophilic polymer layer on the surface of a membrane (flat or hollow fiber) formed, as a rule, from a hydrophobic synthetic polymer. The formation of the surface layer is carried out in various ways: immersion [59], dipping [60], sputtering [61], and the introduction of appropriate additives into the precipitation bath during membrane formation [62,63]. An interesting approach to surface treatment is the plasma treatment of membranes [64].

Each of the above methods has its drawbacks. Thus, the grafting of functional groups is usually a long and multistage chemical process [46,65], which increases the cost of membranes by several times. Membranes obtained by mixing polymers show low stability in operation: a hydrophilic polymer is prone to leaching, while membranes are prone to re-contamination [66]. The incorporation of inorganic particles into the polymer matrix turns out to be ineffective due to the fact that most of them remain “turned off” from the surface separation process, and the application of a surface layer usually leads to a significant decrease in permeability. In this regard, there is no universal approach to the modification of membranes intended for the separation of water-oil emulsions. Each polymer must be considered individually.

### 2.1. Polysulfone, Polyethersulfone

Polysulfone (PSf) and its derivatives, due to its high chemical, oxidative, and thermal stability and good mechanical strength, is one of the most important and common membrane materials [67]. In addition, the high interest in this polymer is explained by the possibility of the extensive modification of membranes based on it [68]. The traditional method of manufacturing PSf membranes is phase inversion in a non-solvent. Dimethylformamide (DMF), dimethylacetamide (DMAA), dimethyl sulfoxide (DMSO), formylpiperidine morpholine, and N-methylpyrrolidone (NMP) can be used as a solvent for preparing casting solutions from PSf [35]. Recently, there has been a tendency to replace traditional toxic solvents with green, environmentally friendly ones—for example, the Rhodiasolv PolarClean solvent [69].

Due to the hydrophobicity of the PSf polymer and its tendency to contaminate, when creating membranes for the separation of water-oil emulsions, various types of modifications are resorted to. One of the most common modification methods is the addition of a hydrophilic polymer to the casting solution. The paper [47] presents the results of studies of the ultrafiltration purification of oil-water emulsions using flat asymmetric membranes from a mixture of polysulfone (PSf) and water-soluble polyvinylpyrrolidone (PVP). The membranes were obtained by pouring a solution of a mixture of polymers of the appropriate composition (Table 1) in N-methylpyrrolidone (NMP) using the phase inversion method. The table shows the characteristics of three types of membranes that differ in the composition of the polymers.

The membrane structure was determined by scanning electron microscopy, and based on the results obtained, the authors draw the following conclusions:

- For the same pressure drop, permeate flow is higher for membranes with greater porosity;
- The optimal pressure drop is 100 kPa, since in this case the best combination of the permeate flow with the level of oil retention is achieved for all membrane options;
- The pore size and overall porosity of the membranes decreased with the increasing content of polysulfone in the casting composition.

The membrane containing 15% PSf showed the maximum (98%) oil retention when processing an emulsion containing 100 mg∙L^−1^ oil at ΔP 100 kPa with an initial permeate flow of 20 L∙m^−2^·h^−1^ (during the first 30 min, the flux decreased by 10%).

A similar modification method is to add inorganic particles rather than a polymer to the polymer solution. This approach is presented in [70], where bentonite, a natural hydroaluminosilicate that actively absorbs water, was used as a means of increasing the hydrophilicity of membranes. The main component of bentonite (60–70% wt.) is montmorillonite Al_2_[Si_4_O_10_](OH)_2_nH_2_O, which is a sheet silicate with an expanding structural cell that effectively absorbs water with the formation of a gel-like suspension. Using this material as a filler, composite polysulfone membranes were obtained. Comparative characteristics of two types of PSf membranes filled with silicon dioxide or bentonite are shown in Table 2. It is obvious that with the use of PSf membranes with bentonite (8% wt.), the efficiency of water removal is almost doubled compared to PSf membranes filled with silicon dioxide.

The work [71] presents the modification of hollow fiber membranes made of polyethersulfone by introducing particles of hydrophilic magnesium dihydroxide into the surface layer of the fiber. The authors obtained membranes with high hydrophilicity: the contact angle of wetting the surface of the original membrane was 69.5°, and that of the modified one was 16.4°. The water permeability of the membranes increased from 39 to 573 L∙m^−2^∙h^−1^∙bar^−1^, and the oil retention reached almost 100%. Changes in membrane morphology were confirmed by electron microscopy data. Another much cheaper membrane modifier is candle soot. The introduction of candle soot (i.e., a source of monodisperse carbon nanoparticles) into the membrane matrix made it possible to significantly increase the resistance to pollution. The membranes showed an efficiency of 99.9% in the separation of oil-water emulsions and a diesel-in-water emulsion permeability of 314 L ∙m^−2^∙h^−1^∙bar^−1^. The flow recovery after flushing was 92% [72].

An interesting way to accomplish such modification of membranes is the treatment of membranes with surfactants. Surfactants are adsorbed on polymers and, depending on the structure or the presence of functional groups in their composition, change the surface properties, imparting greater hydrophilicity/hydrophobicity or oleophilicity/oleophobicity to membranes. For example, PSf membranes were modified in [73] by keeping them in a 0.1% solution of lutensol (a nonionic surfactant consisting of polyethylated linear aliphatic alcohols) at 20 °C and pH 7.0, followed by washing with distilled water. The positive effect of surfactant treatment was to reduce membrane contamination with oil products. Unfortunately, this fact was not quantified and was confirmed only by the absence of oil stains in micrographs taken at a magnification of 1500 times. Another option for the surface treatment of membranes is to apply a thin layer of a hydrophilic polymer. The surface deposition of polydopamine allowed Bryan D. McCloskey and his colleagues to obtain effective membranes based on polysulfone for the separation of water-oil emulsions [74]. Coating polysulfone membranes with cross-linked polyethylene glycol diacrylate made it possible to increase membrane permeability by 400% [75]. Such membranes showed stable performance over 24 h of filtration. 

Data on the efficiency of the separation of various emulsions using membranes based on PSf and PES are presented in Table 3.

Polysulfone does not have highly reactive functional groups; therefore, the main approaches to the hydrophilization of PSf are the addition of hydrophilic water-soluble polymers [47,76] or inorganic particles—for example, bentonite [70], silicon oxide [57], or candle soot [72]—to the casting solution. In addition, Table 3 shows that PSF-based membranes are modified by applying thin selective layers of other polymers—for example, dopamine [74] or cross-linked polyethylene glycol diacrylate [75].

### 2.2. Acrylonitrile Polyacrylonitrile

Polyacrylonitrile (PAN) has good mechanical, film-forming properties and stability in organic solvents such as hydrocarbons and their chlorine derivatives (hexane, toluene, and dichloromethane), alcohols, and mild aprotic solvents [77]. PAN was first obtained and sold by DuPont in the form of a spun fiber in 1941 under the trademark Orlon [78]. Since then, polyacrylonitrile has found wide application in membrane separation technologies [79]. Membranes made from this polymer are well known for the ultrafiltration [80,81,82,83] and nanofiltration of aqueous [78] and organic media [84,85]. Polyacrylonitrile is soluble in most aprotic polar solvents such as DMSO, NMP, DMAA, and DMF [86].

Polyacrylonitrile is obtained by the polymerization reaction of acrylonitrile, but sometimes copolymerization reactions are used to improve the performance properties of materials. It is known that polyacrylonitrile membranes have high water permeability but are unstable to pollution, and, on the contrary, cellulose acetate membranes are resistant to pollution but have low water permeability. Based on these data, the authors of [49] conducted studies on the synthesis of copolymers based on these materials by grafting acrylonitrile to cellulose acetate by radical polymerization. Thus, copolymers containing 6.3, 19.0, and 21.6% of the mas. Acrylonitrile were obtained; however, flat (sheet) ultrafiltration membranes were obtained only from the first two copolymers, since it was not possible to find a suitable solvent for the third when forming the membrane. The membranes were obtained by the method of phase inversion by pouring a spinning solution of copolymers (copolymer concentration 16% wt.) in dimethylformamide, followed by precipitation. The membrane structure was studied by scanning electron microscopy. Experiments on ultrafiltration purification were carried out using a model stabilized aqueous emulsion of vacuum oil with a concentration of 600 to 1800 mg·L^−1^ at a temperature of 25 °C and a pressure drop of 0.1 MPa. The initial flow for composite membranes was 300–350 L·m^−2^·h^−1^ but after 40 min was reduced to 100 L·m^−2^·h^−1^. After flushing the system with a stream of clean water, productivity was restored. Oil rejection was close to 100%.

Zahed Shami and his colleagues used the copolymerization method to create membranes from styrene and acrylonitrile [87]. The authors obtained superhydrophilic membranes with a water contact angle of 0°. Experiments on the separation of water-oil emulsions were carried out using a model mixture of water and petroleum ether, toluene, and hexane (1% vol.) under the influence of gravity or pressure of 0.1 bar. The membranes provided the effective removal of oil products with flows from 1300 to 2100 L·m^−2^·h^−1^.

Sometimes, to modify membranes based on hydrophobic polymers, the addition of a hydrophilic polymer and inorganic particles—for example, oxides or salts of various metals—is used simultaneously. Thus, in [88], a superhydrophilic ultrafiltration membrane made of polyacrylonitrile (PAN) was obtained as follows. The amphiphilic copolymer PF 127 was incorporated into the initial PAN matrix, which is a mixture of copolymers of the following composition: poly(ethylene oxide)-poly(propylene oxide)-poly(ethylene oxide). It therefore possesses superhigh hydrophilicity [89]. In addition, calcium carbonate nanoparticles were introduced into the mixture. The membranes were molded by the phase inversion method from solutions with the following composition: PAN, 9.0; PF 127—1.0; CaCO_3_—0–0.75. The rest is n-methylpyrrolidone. The oil-water separable emulsion contained engine oil (1 mL) and water (1 L). The separation efficiency of the emulsion was 98% and the wetting angle was 20°. The flow of purified water reached a value of 343 L·m^−2^·h^−1^. It should be noted that membranes made of polyethersulfone modified with the same PF 127 copolymer had a larger wetting angle (30°) but lower productivity in terms of removed water (132 L·m^−2^·h^−1^) [76].

Of interest are the works in which the approach of using two types of fillers is implemented—on the one hand, increasing the hydrophilicity, and on the other hand, the strength of the membranes. For example, in [90], the results of studies on the creation of composite membranes based on polyacrylonitrile filled with graphene oxide and silicon dioxide, designed to separate oil-containing emulsions, are presented. The membranes were obtained by electrospinning from a casting composition containing polyacrylonitrile filled with nanoparticles of graphene oxide and silicon dioxide. It was shown by scanning electronoscopy that the fillers are evenly distributed in the structure of the hollow fiber membrane. In this case, graphene oxide is located inside the PAN fiber, and silicon dioxide particles are incorporated into the outer surface. This structure increases the hydrophilicity of the outer surface of the hollow fiber, and the flow of water separated from the emulsion increases from 2600 to 3151 L·m^−2^·h^−1^ when separating the water-oil emulsion.

The presence of reactive nitrile groups in the polymer opens up wide possibilities for their modification—for example, by hydrolysis with the formation of highly hydrophilic carboxyl groups [50]. In [50], PAN ultrafiltration membranes were treated with NaOH solutions at concentrations of 0%, 2%, 5%, and 10% (mass) for 4 h at 30 °C. The resulting membranes were washed with deionized water and treated with 2 M hydrochloric acid at room temperature and finally dried in air with a humidity of 50%. It was found that the optimal concentration of alkali is 10 wt%. As a result, mechanically strong hydrophilic membranes were obtained. The water permeability of the membranes was 2270 L·m^−2^·h^−1^·bar^−1^ at trace (≤10 ppm) concentrations of oil products, and the rejection was R ≈ 99%. Using the same approach to PAN hydrophilization, other authors obtained an efficient composite membrane filled with graphene oxide [91]. Polyacrylonitrile turned out to be a good material for obtaining highly hydrophilic membranes with other functional groups—for example, hydroxylamine groups—providing water contact angles of ≤1 [92].

Data on the separation efficiency of various emulsions using membranes based on PAN are presented in Table 4.

Polyacrylonitrile, unlike polysulfone, has nitrile groups that can be functionalized by alkaline hydrolysis [50], amidoximation [92], or subjected to copolymerization reactions— for example, with acetylcellulose [49] or sterene [87]. In addition, modification with inorganic particles [88,93] is used.

### 2.3. Polyvinylidene Fluoride

Polyvinylidene fluoride (PVDF)—is a semi-crystalline polymer with a repeating unit—(CH_2_-CF_2_)_n._ It is distinguished by high mechanical, chemical, and thermal stability [94]. It is soluble in aprotic polar solvents (NMP, DMF, DMAA), which makes it possible to manufacture membranes from it using the traditional phase inversion technology. Membranes based on it have been known since the 1980s [94]. To date, many methods have been developed for modifying PVDF-based membranes [95].

**Scheme 2:**
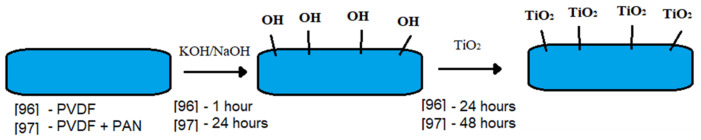
Scheme of the modification of a hollow fiber membrane from PVDF [96] and PVDF + PAN [97].

Chemical grafting of titanium oxide onto the surface of PVDF membranes was carried out in [96]. The order of operations for modifying PVDF membranes is shown in Scheme 2. At the first stage, the hollow fiber PVDF membrane was treated with an alkali solution to replace fluorine ions with hydroxide ions—the dehydrofluorination process. Then, the membrane surface was treated with a suspension of titanium dioxide for grafting oxy and titanium oxide groups. As a result, hollow fiber membranes with a superhydrophilic surface were obtained, capable of separating oil-water emulsions with almost 100% selectivity.

A similar approach was also described in [97], where hollow fiber membranes made from a mixture of PVDF-PAN (contact angle 92.7) were used as the starting material. At the first stage, the fiber was treated in vacuum with microwave argon plasma, followed by immersion in a sodium hydroxide solution to replace chlorine and fluorine atoms with hydroxyl groups. In the second step, the fiber surface was treated with titanium dioxide and a super-hydrophilic fiber surface was obtained, showing a water contact angle of 0.

In another work [98], graphene oxide and titanium oxide were deposited by co-deposition on a PVDF substrate. The use of titanium oxide makes it possible to increase the distance between the layers of graphene oxide, which prevents its compaction and contributes to a more stable permeability over time. Such membranes had an oil-water emulsion permeability of 243 L·m^−2^·h^−1^∙bar^−1^ and a separation factor of 98%.

Another method of chemical surface modification of PVDF is the grafting of short-chain alkylamines and zwitterionization by reaction with 1,3-propanesultone [99]. Such membranes provided 98% separation efficiency of oil-water emulsions even after 10 filtration cycles. PVDF substrates are common in the manufacture of membranes for the filtration of water-oil emulsions. For example, PVDF membranes with nonionic poly(N-acryloylmorpholine) grafted onto its surface [100] are known in the literature. Such membranes were tested in the filtration of an oil-water emulsion at a concentration of 100 mg/liter by varying the emulsion-stabilizing surfactant. The chemical modification of the PVDF surface consists in the grafting of aminosilanes [101].

The work [102] also describes the fabrication of a universal PVDF membrane, which makes it possible to separate emulsions of both types. At the first stage, a polyvinylidene fluoride (PVDF) nanofiber membrane was obtained using electrospinning technology. Then, the membrane surface was treated with alkali, followed by the grafting of polyacrylic acid (PAA) onto it. The resulting membrane of the PVDF-graft-PAA structure can change wettability (hydrophilicity or oligophilicity) depending on pH and the type of aqueous emulsion being separated in the range of wetting angle values from 0 to 149. This ensures the separation efficiency of oil-in-water and water-in-oil emulsions at a level of at least 99%.

Surface physical modification of PVDF membranes can be carried out by applying a thin layer of a hydrophilic polymer. In the work of scientists from China, a layer of a hydrophilic polymer, cellulose, was applied to the surface of PVDF using a “glue” of tannic acid and polyvinyl alcohol [103]. Such a membrane showed high resistance to contamination and retained high values of permeability P = 318 L·m^−2^·h^−1^∙bar^−1^ and separation factors R = 99.7% even after 30 filtration cycles. Another interesting option for the hydrophilic coating of PVDF membranes consists of plant-derived epigallocatechin gallate (EGCG) and silver ion (Ag^+^) [104]. Such membranes showed high permeability to the emulsion of diesel in water, 735 L·m^−2^·h^−1^. Note that the emulsion permeability of the initial PVDF membranes was zero.

Data on the separation efficiency of various emulsions using membranes based on PVDF are presented in Table 5.

Polyvinylidene fluoride has a large number of -F groups, thanks to which it is possible to carry out chemical grafting—for example, by aminosilanes [101], alkylamines [99], and, through the defluorination stage, titanium oxide [96]. In addition, PVDF membranes are modified by applying hydrophilic layers, dopamine [105], cellulose [103], and polymer mixing [107].

### 2.4. Other Commercial Polymers

Of course, the list of polymers that are used to create membranes is not limited to PSF, PAN, and PVDF. There are other hydrophobic polymers on the basis of which researchers create high-performance membranes for separating oil-water emulsions. They are often distinguished by special, additional properties that increase their competitiveness with other membranes. The basis for the choice of membrane technology is often the economic component. Note that the price of these polymers is commensurate with PSF, PAN, and PVDF and is sometimes even lower (Table 6).

When developing membranes for separating oil-in-water emulsions, it is very important to consider their service life. Only those membranes that are able to work for a long time without significant loss of their properties can be considered for their further introduction into industry. A big step in this direction was made by researchers who created modified membranes based on polybenzimidazole (PBI) [109]. The application of polydopamine to the polymer and the inclusion of graphene oxide in the matrix made it possible to achieve a high resistance of membranes to biofouling, which was verified by tests lasting 180 days. The permeability of the membranes for the oil-in-water emulsion was 91 L·m^−2^·h^−1^·bar^−1^, and the efficiency was 99.9%.

Of particular interest are bifunctional membranes, which make it possible to separate emulsions of both types: “water-oil” and “oil-water”. In order to create such membranes, chemical surface treatment or the application of additional polymers is usually used. So, in the work [53], two-sided membranes were created. One side was super-hydrophilic and the other side was super-hydrophobic. The membranes were made on the basis of polytetrafluoroethylene (PTFE). At the first stage, hydrophilic layers of polyaniline (PANI) were formed on both substrate surfaces. Then, a layer of silicon nanoparticles (15 nm) modified with perfluorooctyltrichlorosilane was deposited on one side of the resulting membrane from a solution in toluene, which ensured the superhydrophobicity of the resulting coating. The separation process was investigated using aqueous emulsions containing gasoline, toluene, n-hexane, and lubricating oil. The water flow for gasoline-based emulsions was, for “oil-water”, at least 1300 L·m^−2^·h^−1^, and, for the “water-oil” emulsion, 1800 L·m^−2^·h^−1^. The separation efficiency of such emulsions in both cases and in all cycles was at least 99%. Work [110] shows an alternative method that excludes the use of additional reagents and polymers. Thus, researchers use structure-induced switching of the surface properties of polyurethane acrylate (PUA) membranes. The oleophilic nature of the single layer membranes was switched to an oleophobic state in the dual layer configuration due to the formation of air pockets in the dual layer configuration when exposed to low voltage (500 V). Such membranes have shown their efficiency of 99% and high permeability in the separation of mixtures of oil and water (P = 200–1500 L·m^−2^·h^−1^), as well as their emulsions (P = 250–1000 L·m^−2^·h^−1^) [110].

The attempts of researchers to create combined purification methods that would allow for the fast and efficient separation of large volumes of mixtures of water and oil with high concentrations are important. Most often, such methods use the consistent application of traditional and membrane technologies. However, studies in which the membrane performs a dual function deserve even more attention. For example, in work [111], a nickel-plated polypropylene (PPy) fabric is both the cathode for electroflotation and also provides filtration separation. Such a membrane is capable of separating 50% of a mixture of oil products under the action of gravity alone and has excellent resistance to contamination: FRR = 92–94%. In addition, the membrane also shows its effectiveness in separating an oil-in-water emulsion stabilized with surfactants, Tween-80. In this case, the separation efficiency turns out to be much higher when the emulsion is simultaneously exposed to an electric field and when an electrolyte, Na_2_SO_4_, with a concentration of 20 g·L^−1^, is added to the emulsion.

### 2.5. Natural Polymers

As mentioned earlier, membranes intended for the separation of aqueous emulsions of petroleum products must have maximum hydrophilicity (the value of the wetting angle for water ≤ 5°) and oleophobicity (the value of this indicator ≥ 150), which excludes (or minimizes) the possible contamination with organic components of water-oil emulsions. Cellulose and materials based on it fully meet this requirement [45,49,50,51,61]. In addition to technological adequacy, membranes based on cellulose and its derivatives are in good agreement with the requirements of “green” technologies [112]. Depending on the origin, degree of polymerization, and molecular weight of cellulose, membranes are promising for separating emulsions with various oil content. For example, bacterial cellulose membranes (which are obtained from sucrose in the presence of Acetobacter bacteria and have high mechanical properties) make it possible to purify solutions with very low oil concentrations (only 10–230 ppm [113]), while membranes based on “vegetable” celluloses are commonly used to purify emulsions at concentrations of 500–1000 mg·L^−1^. Sometimes, in order to improve the performance properties of membranes, cellulose is not used in its pure form but rather modified. For example, in [114], nanolayers of poly(N-isopropylacrylamide) (PNIPAAm)-blockpoly(oligoethylene glycol methacrylate) (PPEGMA) were grafted onto the surface of low molecular weight ultrafiltration membranes made of regenerated cellulose. Unfortunately, this led to a decrease in permeability by about 40%; however, at the same time, it made it possible to increase the resistance of the membranes to pollution.

On the basis of cellulose, universal membranes are known that make it possible to separate emulsions of both types: “oil in water” and “water in oil” [115]. This effect can be achieved by creating a membrane with surfaces of opposite types: one side is superhydrophilic and allows you to purify water from oil, and the other side is superhydrophobic, effective in purifying oil from water. In this case, a simple inversion of the membrane makes it possible to radically change its properties.

Of particular interest are membranes that make it possible to purify aqueous media not only from emulsified petroleum products but also from dissolved components—for example, dyes. In [116], a method for obtaining a polyelectrolyte membrane using nanofibrillar cellulose was proposed. The membrane not only separates oil/water emulsions with high separation efficiency (>99%) and high permeability > 11,000 L·m^−2^·h^−1^∙bar^−1^ but also allows for the removal of positively charged dyes with good permeability (>10,000 L·m^−2^·h^−1^∙bar^−1^) and a rejection greater than 98%. In the work of Wanli Lu and his colleagues [117], a nanofiber membrane obtained by electrospinning from deacetylated cellulose acetate, polyvinylpyrrolidone, and Fe compounds makes it possible to purify water from oil products, dyes, and chromium (VI) with an efficiency of over 99%. Multifunctional membranes are sometimes obtained by combining various natural polymers, such as lignocellulose and chitosan [118]. Such membranes can not only purify water from oil products and dyes but can also purify it from the microorganisms *E. coli*, *S. aureus* and *B. subtilis* with an efficiency of 99.97–99.98%, which becomes possible due to the introduction of Ag particles with antibacterial properties.

A natural polymer, chitosan, is most often used not in its pure form but only for the modification of cellulose [118] and synthetic polymers—for example, polyethylene terephthalate [119], polycaprolactone [120], polyvinylidene fluoride [121], and polysulfone [122]. Such a modification makes it possible to increase the hydrophilicity of the membranes, which means to increase their resistance to contamination in the process of separating oil-water emulsions and to increase the stability of indicators over time.

A commercial polymer derived from cellulose is cellulose acetate, on the basis of which it is also possible to obtain effective membranes under ultrafiltration conditions. Cellulose acetate is soluble in DMF, DMAA, DMSO, tetrahydrofuran (THF), acetone, dioxane, and acetic acid, which makes it possible to produce membranes based on it by the phase inversion method [36]. Interesting three-dimensional membranes with a hierarchical structure are known, consisting of cellulose acetate coated with graphene oxide (GO) or layered double hydroxyls (LDH) grafted with sepiolite (SeP). In this work, it is shown that the use of graphene oxide for modification is preferable in comparison with LDH, because such membranes have higher permeability and show a slower decline in fluxes over time [123]. Data on the separation efficiency of various emulsions using membranes based on natural polymers are presented in Table 7.

Natural polymers and cellulose are hydrophilic, so they can be used without additional modification steps [113,124,125]. Sometimes, modifications are resorted to with the help of chemical grafting [128] and the coating of organic [115] and inorganic materials [123]. One of the popular natural polymers used for membrane modification is dopamine [116,129].

### 2.6. Polymers as Thin Selective Layers on Inorganic Support

All of the above polymers, both hydrophobic (PSf, PES, PAN, PVDF) and natural hydrophilic ones, can be used not only as independent membrane materials but also as polymers for depositing thin selective layers on ceramic substrates. On the one hand, due to the properties of the applied polymers, this ensures high selectivity of the resulting membranes. On the other hand, thanks to the highly permeable substrate, the membranes perform well.

A typical experimental algorithm for the preparation and study of ceramic-based composite membranes is presented using the example described in [130]. The composite membrane was prepared as follows. At the first stage, a ceramic flat porous substrate was obtained by sintering the mixture (composition, wt.%: kaolin 75, calcium carbonate 20, boric acid 2.5, and metasilicate 2.5) at a temperature of 900 °C. At the second stage, a composite membrane was molded by immersing the resulting substrate into a 5% solution of cellulose acetate in acetone, followed by exposure at various exposure times at temperatures of 15, 25, and 40 °C and at pH values of the solution of 7–12. The samples obtained were characterized by infrared, electron, and X-ray spectroscopy. The porosity of the resulting membranes was 28–68%, and the pore size was 30 ÷ 47 nm (ultrafiltration range). The object of study was an oil-water emulsion (concentration 100 and 200 mg·L^−1^) prepared from crude oil using ultrasonic stirring to ensure the homogeneity of the emulsion. The degree of oil rejection was 99.6% at an initial concentration of 100 mg·L^−1^. However, it should be noted that this flow is unstable and drops from the initial value of 0.43 m^3^ m^−2^ h^−1^ to a stable one, starting from 25 min ≈ 0.23 m^3^ m^−2^ h^−1^. The same is observed at a pressure drop of 4 bar.

Another approach to the preparation of planar ceramic membranes is presented in [131]. Fouling-resistant ultrafiltration membranes were obtained by grafting on the surface and in the pores of ceramic plates pre-treated at a temperature of 70 °C with a mixture of vinyltrimethoxysilane and vinylpyrrolidone using hydrogen peroxide as an initiator of copolymerization. The average values of the wetting angles of the resulting membranes were about 4–5, which indicates the formation of a hydrophilic layer on the surface and in the pores of the substrate. The separation efficiency of oil-containing microemulsions (initial oil concentration 36,350 ppm, droplet size 18–66 nm) at a filtration temperature of 24 °C was 47–53%.

An interesting variant of obtaining a flat composite membrane with a high surface hydrophilicity is presented in [48]. As a substrate, ammoniated zirconium dicarboxylate (zirconosilicate) was used, a porous material impregnated with a solution of polyamide 66 (5% wt.) and a composition of ZrO-66-NH_2_ with hydrophilic carboxyl and amino groups, which is chemically stable in an aqueous medium. By grafting a polyacrylonitrile membrane onto this material, the authors obtained a composite membrane that provides highly efficient separation of oil-water emulsions, since the membrane surface contains carboxyl and amino groups, which ensure its high hydrophilicity. It is noteworthy that only under the action of gravity (~0.01 bar) can the resulting membrane separate oil-water emulsions with very high efficiency (>99%, with a residual content of organic substances < 10 mg·L^−1^) with a total performance of purified water about 2.1 m^3^ m^−2^ h^−1^. The authors explain such a high selectivity of emulsion separation by the presence of carboxyl and amine groups in the surface layer of the membranes, which are superhydrophilic with respect to the water and superoleophobic with respect to the oil component of emulsions. The same circumstance explains the high resistance of the membranes to contamination by the components of the emulsion being separated. Similar results are presented in [52], which describes the results of a study of oil extraction from process wastewater using a membrane apparatus equipped with tubular ceramic elements, the inner (working) surface of which was treated with a 5% solution of polyamide-66. When cleaning emulsions containing 50, 100, and 200 mg·L^−1^ oil, the degree of extraction reached 99.5%. At the suggestion of the authors [48], such approaches were designated as “decoration” of the membrane surface.

## 3. Membranes Formed by Electrospinning

Electrospinning is a promising method for forming membranes [132,133]. Compared to NIPS, it allows for the obtention of membranes with very high porosity and permeability. In addition, it allows one to control the size and thickness of the pores. For the manufacture of membranes by this method, hydrophobic polymers [134] are most often used—for example, polyethersulfone [135,136], polyacrylonitrile [90,91,93], and PVDF [97,98,102]. For their hydrophilization, they resort to modification with oxides of graphene, silicon, and titanium.

In Table 8, most of the membranes produced by electrospinning from various polymers have very high permeability, estimated at several thousand L·m^−2^·h^−1^ for mixtures of oil and water (50% vol.) and emulsions. The high porosity of the membranes allows for separation without the use of excess pressure—only under the action of gravity. The selectivity of the membranes in this case approaches 100%. These facts testify to the high efficiency of the method of membrane molding by electrospinning.

## 4. The Problem of Comparison of Literature Data

The previous sections of this article provide an overview of the literature on the creation of membranes for the separation of water-oil emulsions. Detailed information is provided on such commercial polymers as PSf, PAN, PVDF, and cellulose. Comparative tables are presented for each of them, which make it possible to identify the most effective methods for modifying these polymers. However, it is worth paying attention to the fact that such a comparison is not entirely correct. Thus, the presence of surfactants and inorganic salts in the composition of emulsions, their concentration, the size of emulsified particles, pH, and the concentration of emulsified petroleum products and their type have a great influence on the stability of emulsions and hence on the possibility of their separation.

In order to evaluate the effectiveness of membranes in the separation of water-oil emulsions, it is necessary to know which oil products are included in their composition. Among the petroleum products used to create model mixtures, there are: crude oil, diesel, kerosene, soybean oil, toluene, hexane, and others. Obviously, due to the complexity of the composition, it is much more difficult to separate the crude oil-water emulsion than the toluene-water emulsion. Researchers often pay attention to this fact by citing the permeability of membranes for various oil products. For example, a PVDF membrane coated with dopamine has a permeability of hexane in water of 558 L·m^−2^·h^−1^, and more than 2 times less for diesel 243 L·m^−2^·h^−1^ [105] (Table 5). A cellulose membrane has a permeability of 7500 L·m^−2^·h^−1^ for a colza oil emulsion in water, and for petroleum ether 15,000 L·m^−2^·h^−1^ [117] (Table 7). Thus, it is more correct to compare the efficiency and permeability of membranes studied using emulsions containing the same petroleum product. However, in the case of complex petroleum products such as oil, diesel, kerosene, and various oils, even such an approximation is conditional due to the possibility of a wide variation in the qualitative composition of petroleum products. Works in which researchers prefer not to indicate the oil product used, calling it “oil” [95,97,100], are practically impossible to use for making any comparisons.

In addition, the concentration of oil in the emulsion has a great influence on the permeability and efficiency of membranes. If this fact is not taken into account, it may seem that the modification of PSF with dopamine [75] is much more effective than the application of cross-linked polyethylene glycol diacrylate [77]. So, in the first case, the permeability of membranes for the soybean oil-water emulsion is 65–70 L·m^−2^·h^−1^, which is an order of magnitude greater than 6 L·m^−2^·h^−1^ for the membrane from [77]. However, let us pay attention to the fact that the concentration of the oil product in [75] is only 135 ppm, while it is 1500 ppm in [77], which can have a decisive effect on membrane permeability. In different works, the concentration of oil products ranges from 10 ppm to 100,000 ppm, or up to 50% wt., vol. When separating oil-water mixtures, membrane permeability usually decreases with increasing oil concentration. This was shown in [113], where the concentration increases from 10 to 230 ppm and the permeability decreases by 30%. In [49], the permeability also decreases by slightly more than 30%, with an increase in concentration from 300 to 1800 ppm. In [47], the permeability of PSf membranes decreases by almost an order of magnitude, with an increase in the concentration of oil products from 100 to 400 ppm. The lack of information on the concentration of the oil product also makes it difficult to make comparisons.

The permeability of membranes for emulsions and their effectiveness are significantly affected by the presence of surfactants. In their absence, the emulsion is less stable, and the membranes show higher permeability. This is well shown in [117], where the permeability for unstabilized emulsions is 3–5 times higher than for emulsions containing Tween 80.

In addition, the properties of membranes are affected by the type of surfactants. Anionic sodium dodecylbenzylsulfonate (SDBS), sodium dodecylsulfonate (SDS), cationic hexadecyltrimethylammonium bromide (CTAB), and neutral polysorbate (Tween 80) are most commonly used in oil-water emulsions. The type of surfactant determines the size of the emulsified particles (Table 9).

Table 9 shows that the effect of surfactants is ambiguous and depends on the concentration of the oil product and the concentration of surfactants, indicated as the ratio “surf/oil”. So, in the work [125], the emulsified particles have the maximum size when they are stabilized with anionic surfactants, and in the work [100], with cationic surfactants. The concentration of surfactants is also a variable parameter of emulsions and can range from 20 [103] to 10,000 [96] ppm.

The particle size indicated in Table 8 is only the predominant size of the emulsified particles. Typically, an emulsion is characterized by the size dispersion of the emulsified particles. For example, in the work [137], particles have sizes from 1 to 1000 microns. 

Another component of emulsions can be inorganic salts [138]. In most works, researchers do not mention the concentration of salts. In the work [106] salt concentrations are NaCl = 50 g·L^−1^, CaCl_2_ = 16 g·L^−1^, MgCl_2_ = 8 g·L^−1^, Na_2_SO_4_ = 2 g·L^−1^, and NaHCO_3_ = 1 g·L^−1^. Salt concentration has no definite effect on membrane properties. Thus, in the work [139], it is shown that the membrane permeability for emulsions is maximum at NaCl concentration = 469 mM, minimum at NaCl concentration = 1711 mM, and average at NaCl concentration = 100 mM. In another work, it was shown that the presence of sodium sulfate Na_2_SO_4_ in the emulsion at a concentration of 20 g·L^−1^ makes it possible to increase the separation efficiency [111].

The efficiency of emulsion separation is also affected by the pH of the solutions. This is often due to the possibility of protonation-deprotonation of the functional groups of the polymer and changes in the surface properties of the polymer. So, in an acidic environment (pH = 4), cellulose membranes have a negative zeta potential, and in an alkaline environment (pH = 11), slightly positive zeta potential [125]. This determines the membrane’s ability to retain emulsified oil and resist fouling. So, due to the electrostatic repulsion of particles in an acidic environment, the membranes best retain emulsions with anionic surfactants, and in an alkaline environment, with cationic surfactants. Sometimes, pH also affects the functional ability of membranes. Thus, membranes from [102] based on PVDF have the ability to “switch” the surface properties depending on the pH of the medium. In a neutral environment, such a membrane is able to purify water from oil, and in an acidic environment, on the contrary—oil from water.

In addition, the conditions for the preparation of emulsions, the methods, the duration of its mixing, and even the instrumentation are very important. Thus, it is known that an increase in the intensity of mixing, an increase in the diameter of the agitator, and a decrease in the diameter of the tank leads to a decrease in the size of the emulsified particles [139]. In addition, mixing time and temperature affect emulsification [140]. Unfortunately, many researchers do not even indicate the conditions for preparing emulsions. Thus, even when preparing an emulsion from the same components with the same concentrations but in different laboratories, it is possible to observe a scatter in the size of the emulsified particles and, hence, in the future, in the permeability and selectivity of membranes. This further complicates the comparison task.

Another important parameter is the “lifetime” of the emulsion before testing. All of the parameters listed in the Table 9 affect the stability of emulsions [141,142], which over time are prone to the aggregation of emulsified particles and to separation. Moreover, aggregation is facilitated by the mechanical effect exerted on the emulsion during filtration. Due to the separation of the emulsion, the oil concentration may change. In [123], it is noted that 5 h after the preparation of the emulsion, the oil concentration is 95% of the initial one. In this regard, it would be more correct to carry out tests with emulsions not freshly obtained but stabilized by time. This will allow for the separation of a stable system that will be minimally prone to particle size changes during filtration.

Thus, despite the large amount of data on the use of different polymers to create membranes, their comparison is a difficult task. A correct comparison of methods for creating membranes and/or their modifications can be carried out by fixing all possible variable parameters, the list of which is given in Table 10, which is hardly possible.

## 5. Conclusions

This article presents a review of literature data on the methods for manufacturing membranes and their modifications based on the commercial polymers PSF, PES, PAN, and PVDF, as well as cellulose and others for the separation of water-oil emulsions. It is noted that there is no universal modification method that would always allow for achieving high results. In addition, the methods of possible modifications depend on the chemical structure of the polymers. For example, thin polymer coatings can be used on any polymer. Chemical grafting is only for polymers with reactive functional groups. Therefore, the functionalization of each polymer must be considered separately.

On the contrary, the methods for obtaining membranes for all of the polymers mentioned above are common. The main method for forming these membranes is the phase separation process, NIPS. The NIPS method has been known for a long time and has reliable methods. However, it is impossible not to pay attention to the electrospinning method. Its advantage over NIPS is the possibility of obtaining highly permeable membranes capable of separating emulsions only under the action of gravity.

The most important conclusion of this review is that all available literature data can be used to search for casting methods and modifications but not to compare them. This is due to the large variability in the composition of emulsions (types of petroleum products, surfactants, salts, their concentrations, pH of the medium, and particle sizes), methods of their preparation on which the permeability and selectivity of membranes strongly depend.

## Figures and Tables

**Table 1:** Comparison of the properties of modified PSf membranes [44].

Membrane	The Composition of the Casting Solution			The Composition of the Membranes % wt.		Average Pore Size, µm	P_H2O_	P_em_	R
	PSf	PVP	NMP	PSf	PVP				
M_1_	10	5	85	66.67	33.33	0.3	90	14–27	79
M_2_	12	5	83	70.58	29.42	0.25	79	16–24	83
M_3_	15	5	80	75.00	25.00	0.17	72	18–20	98

An emulsion was prepared using crude oil in a concentration of 100 mg L^−1^. P_H2O_, P_em_—flow of pure water or emulsion accordingly, L/m^2^ h^1^ bar ΔP = 100 kPa. R is the rejection of oil products, %.

**Table 2:** Comparison of the properties of PSf membranes modified with silicon (Si) and bentonite (bent.) [70].

Membrane Type	Equilibrium Water Content % wt.	Pure Water Flow, L ∙ m^−2^ ∙ h^−1^ bar^−1^ P = 300 kPa	Water Flow during Emulsion Cleaning m^3^ ∙ Pa^−1^ ∙ s^−1^ ∙ m^−1^
PSf	20.8	112	0.97
PSf-Si 8%	60.4	275	2.42
PSf-Si 10%	54.1	291	2.64
PSf-bent. 8%	62.2	612	5.25
PSf-bent. 10%	51.0	439	3.83

**Table 3:** Membranes based on PSf and PES for the separation of water-oil emulsions.

Membrane Material	Oil	C_o-p_	SF	C_Surf_	P_ex_	R	P	No	FRR	Ref.
PSf + PVP	Crude oil	100–400 mg·L^−1^	-	*-*	1–4	79–98	10–70	1	-	[47]
PES + PF 127	Soybean oil	900 mg·L^−1^	SDS	100 mg·L^−1^	1.5	≈100	43–83	1	78–90	[76]
PSf + bentonite	Crude oil	100–400 mg·L^−1^	-	-	3	90–95	112–612	1	-	[70]
PES + SiO_2_	Oil	2000 mg·L^−1^	-	-	3	>98	89–291	1	-	[57]
PES + Mg(OH)_2_	Crude oil	100–1000 ppm	-	-	1	≈100	60–573	1	40–75	[71]
PSf + candle soot	Diesel	2% wt.	-	-	1	≈100	314	6	92	[72]
PSf/dopamine	Soybean oil	135 mg·L^−1^	-	-	10.2	98	65	1	44–99	[74]
PSf/dopamine + PEG						98	70			
PSf/crosslinked polyethylene glycol diacrylate	Soybean/canola oil	1500 ppm	DS 193	150 ppm	10	98	6	1	-	[75]

C_o-p_—concentration of oil product (the unit of measurement is indicated in each specific case); SF—surfactants; P_ex_—excess pressure used to filter the emulsion, bar; R—rejection, %; P—emulsion permeability, L·m^−2^·h^−1^∙ bar^−1^; No —number of cycles; PVP—Polyvinylpyrrolidone; PEG—Polyethylene glycol.

**Table 4:** PAN-based membranes for the separation of water-oil emulsions.

Membrane Material	Oil	C_o-p_	SF	C_Surf._	P_ex_	R	P	No	FRR	Ref.
PAN + CA	Vacuum oil	300–1800 mg·L^−1^	SDS	30–180 mg·L^−1^	1.5	≈100	67	3	80–90	[49]
PAN + styrene	Toluene	1.0% vol	-	-	0	-	2100	1	-	[87]
	Hexane						1900			
	Petroleum ether						1100			
	Toluene		SDS	100 mg·L^−1^			1750			
	Hexane						1300			
PAN + PF 127 + CaCO_3_	Motor oil	0.1% vol.	Tween 80	1000 mg·L^−1^	-	98	343	2	98	[88]
PAN, Modified hydroxylamine	Diesel fuel	1% vol.	SDS	100 mg·L^−1^	0.1	96–98	3806	10	100	[92]
	Petroleum ether						3000			
	Isooctane						2100			
	Trichloromethane						2200			
PAN, Alkali Hydrolyzed	Hexane, petroleum ether, i-octane, hexadecane	10% vol	Tween 80	0.1% wt.	1	99	2000–2270	10	95	[50]

C_o-p_—concentration of oil product (the unit of measurement is indicated in each specific case); SF—surfactants, P_ex_—excess pressure used to filter the emulsion, bar; R—rejection, %; P—emulsion permeability, L·m^−2^·h^−1^∙ bar^−1^; No—number of cycles.

**Table 5:** PVDF-based membranes for the separation of water-oil emulsions.

Membrane Material	Oil	C_o-p_	SF	C_Surf_	P_ex_	R	P	No	FRR	Ref.
PVFD + dofamine	Diesel	10% vol.	SDS	200 mg·L^−1^	0.4	>99	243	3	82	[105]
	Gasoline						331		90	
	Hexane						558		87	
PVDF + vermiculite NP	Compressor cleaning fluids	79.5 mg·L^−1^	-	-	0.7–1.5	90.0	≈100	1	-	[106]
		275.7 mg·L^−1^				93.4	≈50			
PVDF + Cellulose	Lubricating oil	0.2% wt.	CTAB	0.002% wt.	0.9	>99	305	30	80–99	[103]
	Diesel oil						310			
	Sunflower oil						318			
	Pump oil						313			
PVDF + plant-derived epigallocatechin gallate (EGCG) and silver ion (Ag^+^)	Diesel fuel	0.1% wt.	SDS	0.01% wt.	0.5	97	735	5	≈100	[104]
	Kerosene					98	561			
	Soybean oil					98	304			
PVDF + grafted short chain alkylamines	Soybean oil	1 mg·L^−1^	SDS	0.2 mg·L^−1^	0.5–1.5	60–98	3–100	10	>96	[99]
	Silicone oil					70–98	10–190			
PVDF + grafted poly(N-acryloylmorpholine	Oil	100 mg·L^−1^	SDBS	1 mg·L^−1^	1	91–99	50–500	5	25–30	[100]
			Tween-80				100–500		50–60	
			CTAB				400–600		≈50	
PVDF + grafted aminosilanes	Toluene, silicone oil, soybean oil	10% wt.	-	-	1–3	99	7–12	1	-	[101]
Defluorinated PVDF + TiO_2_	Oil	50% vol.	Triton X-100	1% wt.	0	≈100	4–25	3	60–99	[96]
PVDF + PEI + TiO_2_	Hexadecane	7650 mg·L^−1^	SDS	50 mg·L^−1^	0.1 bar	≈100	200–300	5	≈100	[107]
PVDF + g-TiO_2_ + PFDS	Water-in-chloroform	0.9% wt.	Span-80	0.4% wt.	1	>99	2421	5	≈100	[108]
	Water-in-toluene						908			
	Water-in-rapeseed oil						118			
	Water-in-soybean oil						57			
	Dodecane in water	0.15% wt	SDS	0.02% wt.			2542			
	N-hexadecane in water						398			
	Soybean oil in water						314			
	Rapeseed oil in water						443			

C_o-p_—concentration of oil product (the unit of measurement is indicated in each specific case); SF—surfactants; P_ex_—excess pressure used to filter the emulsion, bar; R—rejection, %; P—emulsion permeability, L·m^−2^·h^−1^∙ bar^−1^;No—number of cycles; PEI—branched polyethyleneimine; PFDS—1H, 1H, 2H, 2H-perfluorodecyltri-ethoxysilane.

**Table 6:** The cost of polymers (it is assumed that 1 US dollar = 75 rubles).

Polymer	Approximate Cost, $/kg	Manufacturer (Country)
PAN	2	Haihang industry Co (Jinan City, China)
PSf	15	Molan (dongguang) Plastic Technology (Guangdong, China)
PES	16	Molan (dongguang) Plastic Technology (Guangdong, China)
PVDF	36	AGRU KunststofftechnikGmbH (Bad Hall, Austria)
PBI	2	PBI Performance Products, Inc. (Charlotte, US)
PTFE	17	Hebei Luozheng Technology Co (Hebei, China)
PANI	10	Shandong Ningda Chemical Co (Shandong, China)
PUA	15	ANWIN TECHNOLOGY CO (Tapiei, Taiwan)
PPy	2	Dawn Groupe Co (Shandong, China)

**Table 7:** Membranes based on natural polymers and their derivatives for the separation of oil-in-water emulsions.

Membrane Material	Oil	C_o-p_	SF	C_Surf._	P_ex_	R	P	No	FRR	Ref.
Cellulose	Crude oil	200–1000 ppm	SDBS	2–10 ppm	5	99	6–13	1	60–90	[124]
			CTAB				1–20			
			Tween-80				1–2			
Cellulose on stainless-steel mesh	Octane, hexane, dodecane, toluene	-	-	-	0	>99	15,200–46,500	10	-	[125]
Cellulose	Oil	10–230 ppm	Tween 20	0.5% wt.	1	≈100	173	20	-	[113]
Lignocellulosic biomass + dofamine	Oil	-	-	-	30	68–97	6–28	1	-	[126]
Cellulose paper + nanofibrillar cellulose hydrogel	Hexane	50% vol.	SDS	2500 mg·L^−1^	0	99	90	1	-	[127]
Cellulose + grafted acrylic acid + acrylamide	Hexane	-	-	-	0	98	-	1	-	[128]
Cellulose acetate + dopamine + cellulose nanofibers	Diesel fuel, cyclohexane, tolulu, petroleum	1% vol.	SDS	100 mg·L^−1^	0.9	99	149–4525	5	90	[116]
Cellulose + PVDF + SiO_2_ nanocomposite	1,2-dichloroethane	3.21% vol.	Tween 80	0.45% vol.	0	95	-	10	-	[115]
	Toluene	3.21% vol.		0.45% vol.		90				
	Hexane	3.21% vol.		0.45% vol.		92				
	Chloroform	1.64% vol.		0.2% vol.		90				
Lignocellulose + chitosan + Ag + TiO_2_	Chloroform	1.6% vol.	-	-	0	98	765	7	-	[118]
Cellulose acetate + GO + layered double hydroxides	Decane, Marcol 52 white oil	1000 mg·L^−1^	-	-	1	98	200–1300	6	50–90	[123]

C_o-p_—concentration of oil product (the unit of measurement is indicated in each specific case); SF—surfactants; P_ex_—excess pressure used to filter the emulsion, bar; R—rejection, %; P—emulsion permeability, L·m^−2^·h^−1^∙ bar^−1^; No—number of cycles; GO—graphene oxide.

**Table 8:** Membranes formed by electrospinning for the separation of oil-in-water emulsions.

Membrane Material	Oil	C_o-p_	SF	C_Surf_	P_ex_	R	P	No	FRR	Ref.
PAN, Alkali Hydrolyzed + Graphene Oxide	Lubricating oil	0.1% wt.	-	-	0	99	1500–3500	4	99	[91]
PAN+ amidoximated SiO_2_	Chloroform	1% vol.	SDS	100 mg·L^−1^	0	98	1250	1	93	[93]
	Dichloroethane					99	1500			
	n-hexane					96	2750			
	Diesel					97	2200			
Nanofibers from deacetylated cellulose acetate + PVP + Fe compounds	Petroleum ether	1% vol	-	-	0	99	15,000	5	-	[117]
	Cyclohexane						12,000			
	Toluene						10,000			
	Silicone oil						12,500			
	Colza oil						7500			
	Petroleum ether	1% vol	Tween 80	100 mg·L^−1^	0	99	3500	5	-	
	Cyclohexane						3000			
	Toluene						3300			
	Silicone oil						3500			
	Colza oil						2800			
PAN+ graphene oxide + silicon oxide	Lubricating oil	10% wt.	SDS	2000 ppm	0	98–100	2600–3151	5	94	[90]
PVDF + PAN, treated NaOH + TiO_2_	Oil	50% vol.	-	-	0	73–100	10,000–30,000	10	≈100	[97]
PVDF +PVP + TiO_2_ + graphene oxide	Hexadecane	1% wt.	SDS	0.015 wt.	0	98	243	1	-	[98]
PVDF + polyacrylic acid	Hexane	50% vol.	-	-	0	99	9600	5	-	[102]
Cellulose nanofibers	Hexane, petroleum ether, kerosene	50% vol.	-	-	0	>99	120,000	10	-	[129]
Cellulose + graphene oxide	Hexane, toluene	50% vol	-	-	0	≈100	960	10	98	[61]
PVS + SiO_2_	Kerosene	2% vol.	Tween 80	2000 mg·L^−1^	0	96	1450	5	99	[137]

C_o-p_—concentration of oil product (the unit of measurement is indicated in each specific case); SF—surfactants; P_ex_—excess pressure used to filter the emulsion, bar; R—rejection, %; P—emulsion permeability, L·m^−2^·h^−1^; No—number of cycles.

**Table 9:** Effect of surfactant type on the size of emulsified oil particles.

Surfactant Type	Size of Emulsified Oil Particles, nm	
	*C*_oil_ = 200 ppm, Surf/oil = 1:100 [125]	*C*_oil_ = 100 ppm, Surf/oil = 1:10 [100]
Anionic (SDBS)	700–2500	260–300
Cationic (CTAB)	200–300	350–390
Non-ionic (Tween 80)	200–230	210–245

**Table 10:** The composition of oil-water emulsions and methods for their preparation.

Variable Parameter	Possible Options
Composition of emulsions	
Type of oil product	Crude oil, diesel, kerosene, soybean oil, canola oil, vacuum oil, pump oil, sunflower oil, lubricatting oil, petroleum ether, toluene, hexane, hexadecane, motor oil, isooctane, chloroform, dichliroethane, gasoline
Oil product concentration	10–100,000 ppm
The presence of surfactants	SDS, SDBS, CTAB, Tween-80, Span 80, Trinon X100
Surfactant concentration	20–10,000 ppm
Presence of inorganic salts	NaCl, CaCl_2_, MgCl_2_, Na_2_SO_4_, NaHCO_3_
Salt concentration	1–20 g·L^−1^
pH	1–14
Emulsified particle size	200–1,000,000 nm
Emulsion preparation parameters	
Mixing intensity	350–15,000 rpm
Mixing time	5–360 min

## Data Availability

The data presented in this study are available upon request from the corresponding author.
